# Students’ perceptions of anatomy across the undergraduate problem-based learning medical curriculum: a phenomenographical study

**DOI:** 10.1186/1472-6920-13-152

**Published:** 2013-11-19

**Authors:** Esther M Bergman, Anique BH de Bruin, Andreas Herrler, Inge WH Verheijen, Albert JJA Scherpbier, Cees PM van der Vleuten

**Affiliations:** 1 Department of Educational Development and Research, Maastricht University, Maastricht, The Netherlands; 2 Department of Anatomy, Radboud University Medical Centre Nijmegen, Nijmegen, The Netherlands; 3 Department of Anatomy & Embryology, Maastricht University, Maastricht, The Netherlands; 4 Medical Student, Faculty of Health, Medicine and Life Sciences, Maastricht University, Maastricht, The Netherlands; 5 Dean, Faculty of Health, Medicine and Life Sciences, Maastricht University, Maastricht, Netherlands

**Keywords:** Anatomy, Basic sciences, Curriculum, Education, Knowledge, Learning, Problem based learning

## Abstract

**Background:**

To get insight in how theoretical knowledge is transformed into clinical skills, important information may arise from mapping the development of anatomical knowledge during the undergraduate medical curriculum. If we want to gain a better understanding of teaching and learning in anatomy, it may be pertinent to move beyond the question of *how* and consider also the *what, why* and *when* of anatomy education.

**Methods:**

A purposive sample of 78 medical students from the 2^nd^, 3^rd^, 4^th^ and 6^th^ year of a PBL curriculum participated in 4 focus groups. Each group came together twice, and all meetings were recorded and transcribed verbatim. Data were analysed with template analysis using a phenomenographical approach.

**Results:**

Five major topics emerged and are described covering the students’ perceptions on their anatomy education and anatomical knowledge: 1) motivation to study anatomy, 2) the relevance of anatomical knowledge, 3) assessment of anatomical knowledge, 4) students’ (in)security about their anatomical knowledge and 5) the use of anatomical knowledge in clinical practice.

**Conclusions:**

Results indicated that a PBL approach in itself was not enough to ensure adequate learning of anatomy, and support the hypothesis that educational principles like time-on-task and repetition, have a stronger impact on students’ perceived and actual anatomical knowledge than the educational approach underpinning a curriculum. For example, students state that repetitive studying of the subject increases retention of knowledge to a greater extent than stricter assessment, and teaching in context enhances motivation and transfer. Innovations in teaching and assessment, like spiral curriculum, teaching in context, teaching for transfer and assessment *for* learning (rewarding understanding and higher order cognitive skills), are required to improve anatomy education.

## Background

For many clinical specialties, a good knowledge of anatomy is indispensable to ensure safe and efficient clinical practice [1,2]. Together with physiology and biochemistry, anatomy is one of the basic sciences that are to be taught in the medical curriculum [3]. In Problem-based Learning (PBL) curricula, basic sciences are introduced simultaneously, so-called *horizontal integration*, and basic science instruction is integrated with clinical science instruction, so-called *vertical integration*[4]. In order to achieve this, basic and clinical sciences are not presented in separate courses but in integrated themes [3]. PBL aims to promote active engagement of students in their own learning by stimulating constructive, self-directed, collaborative and contextual learning and by using (patient) problems as triggers of learning [5]. Several meta-analyses have shown that PBL and traditional curricula *do not differ in any way* with regard to students’ performance on tests of basic science and clinical knowledge [6-8], and studies of students’ basic science and anatomy knowledge have revealed neither benefits nor drawbacks of PBL curricula compared to traditional ones [9-12]. Nevertheless, it has been pointed out that there is a discrepancy between students’ *actual* and *perceived* knowledge of basic sciences [12-14]. A questionnaire among students in a PBL curriculum at the start of the clinical phase showed that only 18.5% thought their basic science knowledge was sufficient, with most deficiencies being perceived in pharmacology and anatomy [13]. Interestingly, students’ views appeared to change with experience. When Prince et al. [14] asked anatomists, clinicians, beginning clerks and recent graduates to set a pass/fail standard for an anatomy test at the start of clinical training, the highest standard (failing 64%) was set by the students, but the recent graduates were by far the most lenient judges (failing only 26%), while clinicians and anatomists failed 58% and 42%, respectively. The researchers suggested that the leniency of the recent graduates might be attributable to insight gained during clinical experience into the level of knowledge actually required in clinical practice and to their awareness of how much they had learned during the clerkships. It remains unclear, however, why, at the start of clinical training, students should feel so insecure about their knowledge and set such high standards for themselves.

It has been hypothesised that students’ perceived and actual knowledge is less determined by the general educational approach of the curriculum than by educational strategies like time- on-task, repetition and teaching in context [12,15]. There is, however, no research evidence to support this, and a recent literature study seeking empirical evidence for factors affecting students’ anatomical knowledge unfortunately failed to yield any meaningful conclusions due to the generally poor quality of studies [16]. Although quite varied, the current literature on anatomy education seems to focus on *methods of teaching*[17]. An example is the ongoing debate about cadaver dissection as an educational tool [2,18,19]. Despite studies showing that a dissection course is not a uniform learning experience [20], that students do not always rate dissection as the most useful educational tool [21] and that they do not consider all aspects of anatomical education to be meaningful [22], the debate has remained unresolved so far. However, perhaps we might learn from the debate that if we want to gain a better understanding of teaching and learning in anatomy, it may be pertinent to move beyond the question of *how* and consider also the *what, why* and *when* of anatomy education. In search of an answer to these questions, we explored the perceptions of students in a PBL curriculum regarding their anatomy education and knowledge.

## Methods

### Theoretical framework

Expecting that the perceptions of students at different stages of the undergraduate curriculum would reflect a process of complex, unstable, non-linear change in medical education [23], we opted for a constructivist paradigm in designing the study. The constructivist paradigm is grounded in relativist ontology and subjectivist epistemology, which assume the existence of multiple and sometimes conflicting realities that are socially and experientially based, dependent on individuals [24] and that different people experience the same world in different ways.

We analysed the data using a phenomenographical approach, aimed at describing, analysing and understanding: 1) *experiences or perceptions* of a phenomenon (anatomy) and 2) the *different ways* in which a phenomenon is perceived and understood [25,26]. Because group processes were expected to stimulate students to explore and articulate their perceptions and push the boundaries of the discussion further than would be likely in one-on-one interviews [27], we gathered data in sessions of focus groups composed of students at different stages of the undergraduate curriculum.

### Setting

**Figure 1:**
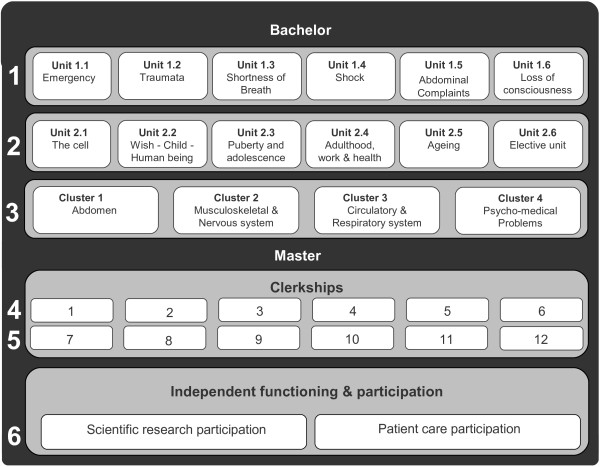
**Curriculum map of the PBL curriculum of Maastricht University at time of this study.** The first and second year are each divided in six thematic units, the third year is divided in four clusters. The fourth and fifth year are devoted to twelve different clerkships. Half of the sixth year is dedicated to participation in a research project the other half is dedicated to in patient care (extensive clerkship).

The study was conducted in the PBL curriculum of Maastricht University, the Netherlands (see Figure 1). At the time of this study, years 1 and 2 consist of six thematic units of approximately six weeks, with few real patient encounters. Paper cases are the starting point for learning in groups of around ten students and a tutor, and knowledge is assessed by 80–120 multiple choice questions in end-of-block tests. In the four systems-based clusters of year 3, the paper cases are replaced by patient contacts in outpatient clinics [28], and knowledge is assessed in oral exams and written essay questions. The anatomy of the thorax, abdomen, pelvis, musculoskeletal system and nervous system is taught in years 1 and 2, and revisited in year 3. In the first three years, anatomy is taught in tutorial groups, during lectures and in the dissection room with models, prosected cadavers and – for the musculoskeletal system - surface anatomy. Years 4 and 5 are devoted to clinical clerkships in twelve disciplines, with formal anatomy instruction being included in only the ophthalmology clerkship and ENT clerkship. In year 6 students undertake an eighteen-week research clerkship and an eighteen-week clinical clerkship with more independence and responsibility for patient care than in the preceding years. Throughout the curriculum, anatomy is also assessed in OSCE’s in years 1, 2, 3 and 5 and four progress tests each year. For a detailed description of the curriculum see Van Berkel et al. [29].

### Ethical approval

Although the Dutch Ethical Review Board informed us that non-patient-related research was exempt from ethical review, we obtained written informed consent from all the participants.

### Participants

To ensure a wide variety in clinical experience, medical students in years 2, 3, 4 and 6 were invited by email to participate in two focus groups. The students in year 2 had just finished year 1, so they had had substantial anatomy education but no real patient encounters. The students in year 3 had just started and where on the brink of going into a series of real patient encounters. Year 4 and year 6 students would have had substantial real patient encounters. We selected students who would be able to attend both sessions, had completed at least half of the clerkship rotations, including the surgical clerkship (year 4) or all clerkships (year 6). The focus groups were scheduled to prevent interference with educational activities and clinical work. Participation was voluntary, sandwiches were available at sessions and students received a small fee for each hour of participation. The number and sizes of the focus groups were arranged to accommodate all volunteers. Participating students of the same year were randomly divided into two groups, in other words, the students from different years were *not* mixed in the groups.

A total of 78 students volunteered to participate (Table 1). With a range of 17–24 students from each year, and an average of 320 students per cohort, they represent about 6% of their population. Of the total number of participants, 75.6% was female, a slight overrepresentation compared to the actual figure of 60-70% per cohort. None of the volunteers were resit students. A T-test comparing Z-scores and percentile scores on progress tests only showed a significant difference in year 4 (p = 0.037, equal variances assumed) which turned out to be attributable to two students being in the top ten of their class.

**Table 1:** Descriptives of focus group participants

		**Focus group number**	**Number of participants**	**Age (years)**		**Gender**	
				**Mean ± SD**	**Range**	**Male**	**Female**
**Junior students**	**Year 2**	Group 1	12	19.5 ± 1.0	19-22	2	10
		Group 2	12	19.1 ± 1.1	18-22	1	11
	**Year 3**	Group 3	10	20.3 ± 0.9	19-22	3	7
		Group 4	9	20.2 ± 0.4	20-21	4	5
**Senior students**	**Year 4**	Group 5	8	21.7 ± 1.0	21-23	1	7
		Group 6	9	24.2 ± 4.0	22-34	5	4
	**Year 6**	Group 7	10	24.3 ± 1.6	23-28	1	9
		Group 8	8	24.8 ± 1.0	23-26	2	6

### Procedure

The two-hour focus group sessions were two weeks apart, so the total duration of the focus group sessions were a maximum of 2x2 hours. The objective of the second session was to elaborate on, or further clarify, statements or discussions that originated in the first session. Students received a written summary of the first session to stimulate elaboration and discussion in the second session. If we had felt that saturation was not reached after these 2x2 hours, we would have scheduled more session. However all groups did not even need the complete 2 hours of the second session to reach saturation and extra session where therefore not necessary. AS moderated the focus groups, guided by a topic list which was iteratively adjusted based on what emerged during the sessions.

### Data analysis

The discussions were audio-recorded and transcribed verbatim. The resulting sixteen transcripts were analysed using template analysis, described by King [30] as a technique for organising (the relationships between) themes emerging during the analysis of (large sets of) textual data, which is particularly effective for comparing the perspectives of different groups.

The transcripts were divided among three researchers (EB, AH and IV), who identified and entered themes and subthemes in individual templates, combining these, after discussion, into one general preliminary template. The researchers sometimes had different views on the division of (sub) themes, but these disagreements were resolved through discussion. Next, they created codes for each theme and subtheme reflecting students’ relevant quotations, and entered these into the preliminary template after discussion. Careful reading and re-reading of the transcripts resulted in the final template (for an excerpt see Table 2) that was used to code all the transcripts. ATLAS.ti 6.0 software (http://www.atlasti.com) was used for the analysis. Disagreements during coding were few, mainly concerning the attribution of multiple codes to one piece of text, and were discussed until consensus was reached. After the coding, the data were organised by themes to enable comparison amongst the groups. EB, AH and IV met several times to discuss and organise students’ perceptions relating to the themes, focusing on emerging similarities and differences amongst the groups. To minimize bias in interpreting the data by EB and AH being anatomy teachers and IV being a student, frequent meetings were held with educationalists CV and AS to discuss the preliminary results.

**Table 2:** Excerpt of final coding template

**Theme**	**Codes**	**Subtheme**	**Codes**	**Subsubtheme**	**Codes**
A.0 Learning anatomy	A.0.1 Rote learning	A.2 Importance of repetition	A.2.1 Importance		
	A.0.2 Lot of work		A.2.2 Scaffolding		
	A.0.3 ‘Fun’				
	A.0.4 Difficult				
	A.0.6 Boring				
		A.3 When is knowledge best retained?	A.3.1 In year 1-3		
			A.3.2 In year 4-6		
		A.4 Motivating factors to learn anatomy		A.9 Assessment	A.9.1 Assessment
				A.12 Patient encounters	A.12.1 Patient encounter
				A.13 PBL	A.13.1 PBL
		A.5 (In)security about anatomical knowledge	A.5.1 During assessment		
			A.5.2 Question of others		
			A.5.3 Insecurity in general		
			A.5.4 Not insecure		
B.0 Anatomy education		B.4 Laboratory sessions		B.24 Dissection room student-teacher ratio	B.24.1 Not enough guidance
					B.24.2 Improvement by more guidance
					B.24.3 Student-assistants
					B.24.4 Improvement by more classical explanation
					B.24.5 Improvement by smaller groups
				B.19 Anatomical learning goals	B.26 When anatomical learning goals are discussed
					B.27 When anatomical learning goals are not discussed
		B.8 The anatomy curriculum	B.8.1 Anatomy curriculum		
		B.9 Amount of detail	B.9.1 Amount of detail		
		B.10 Shortcomings of current anatomy curriculum	B.10.1 Connecting regions		
			B.10.2 Medical imaging		
			B.10.3 Clinic		
C.0 Assessment of anatomical knowledge	C.1 Methods				
	C.2 Assessment in PBL				
D.0 Relevance of anatomical knowledge	D.0.1 Basic science				
	D.0.2 Clinic				
	D.0.3 Communication				
	D.0.4 Assessment				

## Results

Students’ perceptions appeared to centre around five major topics: 1) motivation to study anatomy, 2) the relevance of anatomical knowledge, 3) assessment of anatomical knowledge, 4) students’ (in)security about their anatomical knowledge and 5) the use of anatomical knowledge in clinical practice (for a summery, see Table 3). The results for each topic are presented with illustrative quotes. For the sake of anonymity, the students are identified by unique codes (‘student 6d’ refers to ‘student d’ in group 6 (Table 1)). When perceptions were similar, second-year and third-year students are referred to as junior students and fourth-year and sixth-year students as senior students.

**Table 3:** Final main topics

**Final main topics**	
**Topic 1**	**Motivation to study anatomy**
	Prime motivating factor to study anatomy is clinical exposure: preferably one-to-one (supervised) contact with a real patient, but simulated patients also work well for junior students. PBL is not enough incentive to study anatomy. Without putting anatomy education in context (bringing the patient to the dissection room), learning anatomy is still perceived as boring and depending a lot on rote learning and self-discipline.
**Topic 2**	**Relevance of anatomical knowledge**
	Perceived relevance of anatomy and perceived importance are not synonymous for most students. For junior students relevance is strongly connected to the severity in which a subject assessed. Senior students describe realisation of the relevance of anatomical knowledge only being reached after extensive clinical exposure.
**Topic 3**	**Assessment of anatomical knowledge**
	While students suggest stricter assessment to acquire more anatomical knowledge, senior students suggested that more repetition is necessary to promote knowledge retention. They furthermore acknowledged that repetition also motivates them and increase possibilities of scaffolding.
**Topic 4**	**Students’ (in)security about anatomical knowledge**
	The junior students are very insecure because they feel that they have not mastered everything in the curriculum before their clerkships and expect to get into trouble during patient encounters because of that. Senior students are able to put these feelings in perspective, understanding that these feelings were only natural when students had not yet had any clinical experience. However, the question whether they should master everything in the curriculum led to an unresolved considerable debate.
**Topic 5**	**Use of anatomical knowledge in clinical practice**
	As a clerk students did not encounter any real problems with not knowing all anatomical details. Senior students specifically noted anatomy being taught by region as a specific shortcoming of PBL. They felt that as a result, they did not form a clear coherent picture of the whole human body. This was especially felt when knowledge of blood vessels or nerves was concerned, or when interpreting medical imaging.

### Motivation and stimulation

There was a general feeling among junior students that anatomy was boring. They associated it with having to memorise vast amounts of factual knowledge, requiring more self-discipline than understanding. In marked contrast to the other basic sciences, physiology in particular, anatomy was perceived as learning the names of structures without any attention to how structures were related to clinical signs and symptoms. Senior students recollected this feeling from their earlier years.

“There is nothing to understand, it is just factual knowledge: what is this structure and what is that structure?” (student 1i, year 2).

“[…] physiology, pharmacology and so on are strongly interrelated as regards mechanisms, […] But anatomy, well it’s just cramming. There is nothing to understand about anatomy.” (student 3b, year 3).

The prime motivating factor to study anatomy was clinical exposure. For junior students this could be an encounter with a simulated patient, talking with a real patient in the small group or watching an operation from behind a window. But the strongest stimulus by far was one-to-one (supervised) contact with a *real* patient. Students were very much concerned not to lose face, and real patient encounters also appealed to their sense of responsibility and accountability.

“For, if you don’t know the answer in a test, no-one will blame you. But if you have not prepared and you are facing a doctor, there comes a moment that you just feel rather stupid when you don’t know the answer.” (student 2f, year 2).

“A real patient is indeed more of an incentive, […] this patient said to us ‘I want to ask you a question because I do not understand why the tingling is in these four fingers and not in the other one’ […]. We explained that it was because this nerve was only for those four fingers, but if you had not learnt that, you would really have been stuck for an answer.” (student 3c, year 3).

It was generally agreed that PBL did not offer enough incentive to study anatomy. The paper cases did not stimulate students to learn about anatomy nor to see its relevance. The general opinion was that, for various reasons, it was common for groups to ignore the learning goals relating to anatomy, limiting their opportunities to practise the application of theoretical knowledge to clinical cases. Students suggested that anatomy instruction, both lectures and laboratory sessions, could be made more effective by providing context, for example by pointing to links with radiology or pathology. Students also asked for more guidance in laboratory sessions. To deal with potential staffing problems, they recommended the use of student assistants. It was not so much explanation they were after, although some of them would not mind, but coaching by an enthusiastic teacher with a good idea of their prior knowledge who asked questions and ‘told stories’ showing how anatomy could be useful in understanding and memorising pathological signs and symptoms.

### Relevance versus importance

There was general awareness among the students of the relevance of anatomy to numerous aspects of their future professional practice:

“You need it for diagnosis; you need it for physical examination. For hand-over to colleagues, for record keeping, for writing letters, in fact for understanding how certain processes work, why patients are ill and what should be done about it.” (student 7i, year 6).

As for the junior students, despite their acknowledged awareness of the relevance of anatomy, their notion of relevance was not commensurate with the importance they accorded to anatomy. In other words, they did not give it priority over other study-related and personal activities. Perceived importance of a subject seems to be strongly connected to the severity in which it is assessed; the impact of the subject on assessment results. Perceived relevance of anatomy and perceived importance are not synonymous for these students, and stricter assessment is a frequently mentioned method as an incentive for them to study harder for anatomy (or any other subject).

The full realisation of the relevance of anatomy - its intrinsic value in medicine - came only after extensive clinical experience:

“It was the same for me [insight] that in the clerkships I suddenly thought, hey, at this point it would have been really useful if I had studied a bit more” (student 8f, year 6).

“It is quite easy to survive [the first years] without anatomy […]” “[…] but, [that] is a bad thing, because basically you need anatomy, it is just that when it is taught you don’t realise that, or you don’t want to […]” (student 8e, student 8b, year 6).

Two educational experiences in particular had confronted the sixth-year students with the relevance of anatomy. The neurology clerkship was mentioned by many students:

“[…] I am doing neurology now and there you discover that every diagnosis, everything comes down to anatomy in the end and how things run and work, that is really awfully important.” (student 7g, year 6).

The second experience was the senior clinical clerkship in year 6. Their increased independence and responsibility for patients meant that students had to rely on their own (anatomical) knowledge to a far greater extent than at any time before.

### Assessment versus repetition

A drawback of PBL, according to the students, was integrated assessment, which was particularly detrimental to their learning of anatomy, because it enabled them to pass tests without answering any anatomy question correctly. The strong association between assessment and the perceived importance of anatomy was underscored by students uniformly calling for a more stringent assessment process. A huge amount of focus group time was taken up by discussing ideas for minor adjustments and new methods of assessment. An interesting observation was made by senior students, who noted that, although a more stringent assessment procedure might make them study harder, it would not necessarily promote *retention* of anatomical knowledge. Retention was thought to benefit more from revisiting topics on more than two occasions in the course of the curriculum.

“[…] for anatomy remains a subject […] you really have to repeat, and I mean, for you can study it very thoroughly once and teaching may have been good and it may be assessed, but I think you are bound to forget it after a year, […] you really should look at it again after a year then it will stick in your mind much better […]” (student 6b, year 4).

Both junior and senior students suggested that anatomical topics should be presented with less detail the first time. Junior students arrived at this suggestion by observing that having to memorise a long list of structures was such a daunting task that it was more likely to demotivate them than to encourage them to study:

“Well, as I see it all the time, those arteries, those names and those nerves. I just feel like no, I will never be able to remember all of that. And then I don’t even begin to study.” (student 3h, year 3).

The senior students suggested that the amount of detail should increase with each repetition of a topic. They felt that currently anatomy education was using a ‘one shot approach’ , presenting anatomy topics with all the major and minor structures (the latter being ‘the details’) in one long session. It was suggested that multiple shorter sessions, preferably spread over (more than) the first three years of the curriculum, would be more effective. The first session might cover the major structures (‘the basics’) with more detail being added in subsequent sessions. Students argued that in this way they would learn less per session, but would likely remember more eventually. It was also argued that they would be more motivated to study the same topic again when, on revisiting it, they found they already knew something about it.

“Repetition is really a stimulus. Oh, I already know that, oh but not that.” (student 5g, year 4).

### Insufficiency and insecurity

The vast majority of junior and senior students alike believed that their anatomical knowledge was deficient. They had mastered the broad outlines, but not all the important details. As a result, junior students anticipated problems in the clerkships, based on the assumption that the details were important since they were included in the curriculum. This perceived lack of knowledge made them feel uncertain, and students came up with a variety of ideas for how they might remedy their deficiencies in the future.

Senior students remembered these feelings of uncertainty, but were now able to put them into perspective, understanding that these feelings were only natural when students had not yet had any clinical experience.

“I’ve had different phases, at first I really thought my knowledge is really awful, also for anatomy, and now I am gradually getting to the stage of well perhaps it may be alright after all […]” (students 7a, year 6).

Yes, for I also sort of think well it will be alright, […] yes after all I am only a student […], I mean we should not be thinking that we are all doctors already, and aim so high, that doesn’t make things better” (student 5b, year 4).

The students seemed to be in two minds, however. On the one hand, they were aware that the physicians (residents) they worked with did not know everything about anatomy (just a lot about their own specialty), which reassured them that they did not have to know everything. On the other hand, however, they felt that, since they were training to become a medical doctor and not a specialist, they should have a sound general knowledge of anatomy. This was an issue of considerable debate among the senior students, which remained unresolved.

### Anatomical knowledge in practice

Obviously, the senior students were the only ones who were able to comment on the application of anatomical knowledge in clinical practice. In their experience, less than detailed knowledge did not cause major problems:

“It is not as if in the clerkships I have messed up completely, not at all, but what I find is that you notice, well things are not going quite as smoothly as they should.” (student 5c, year 4).

When discussing the generally felt need for more basic science knowledge, students frequently referred to neuroanatomy or structures extending across body regions, like nerves and blood vessels:

“[…] blood vessels and nerves. These are really the most important things you should see in their entirety […]” “Yes, you would see them run into the foot, but you had no idea where they had branched off” (student 5c & student 5b, year 4).

According to the students, a specific shortcoming of PBL was that anatomy was taught by region, sometimes with months between different regions. As a result, they did not form a clear coherent picture of the human body. Even some of the junior students identified this problem and its clinical consequences:

“And I just thought it strange, you start with the ankle and then, I don’t even remember the order in which we did it, but you really moved from one place to the other […]” (student 7c, year 6).

“One time on a scan you saw something down in the lung and then it turned out to be the stomach coming through the diaphragm. And I would never have thought of that, because to me thorax and abdomen are just two totally unrelated things.” (student 1f, year 2).

Students also mentioned problems applying theoretical knowledge in the clinical context, such as memorising, retrieving and then using knowledge of the musculoskeletal system when examining and diagnosing a patient or interpreting X-rays, CT scans or MRIs:

“[…] what we need in clinic for there they say, yes just take a look at that scan. And then I think like, well, what is the abnormality here, yes I see a shadow there, wow, yes no that is a, that is just an artery, yes sorry, that is …” (student 6d, year 4).

## Discussion

Mapping the development of anatomical knowledge over the continuum of medical training may yield valuable information about how theoretical basic science knowledge transforms into clinically applicable skills and practices [22]. The results of the present study indicate that at different stages of a PBL curriculum students have different perceptions of anatomy education and anatomical knowledge. We will discuss the results in light of the literature to arrive at implications for several areas of anatomy instruction.

### Motivation

Students’ general awareness of the relevance of anatomy is consistent with results reported by Moxham & Plaisant [31]. For junior students, however, relevance appeared to be *dissociated* from the importance of anatomy in terms of study effort: they needed assessment as an incentive to make them study. Assessment driving learning is a well known phenomenon in education [32,33]. An interesting perception was voiced by senior students who argued that stricter assessment might have motivated them to study harder, but would *not* necessarily lead to better retention of knowledge.

Test-directed studying can be regarded as a sign of *external* motivation. Strong *internal* motivation, according to the students, arose from contact with real patients. The introduction of such encounters in the preclinical phase of a PBL curriculum has been described extensively (e.g. Dammers et al. [34] Diemers et al. [35] and Diemers et al. [28]). A study by Takkunnen et al. [36] showed that the introduction of a real patient in a preclinical first-year anatomy curriculum, in addition to paper cases, did enhance motivation, understanding of learning objectives and confidence for future patient encounters, but failed to improve learning outcomes (scores on the course exam). As the implementation and integration of real patient contacts in medical curricula is too complicated to be embarked upon lightly [35], further research should investigate how real patient contacts can have a positive impact on learning outcomes.

In their Self-Determination Theory (SDT), Ryan & Deci [37] described a continuum of *extrinsic* motivation, ranging from impoverished forms of motivation to more active, agentic states. They suggested that students could be stimulated to attain a more active state of extrinsic motivation by fostering a *feeling of competence* within students. According to SDT, students are more likely to adopt an extrinsic goal as their own goal, when they are offered optimal challenges and given relevant feedback. This mechanism appears to resonate with remarks of the students in the focus groups that they would be more motivated to study a topic when it was presented iteratively, because they would recognise the topic and feel they already understood some of it. SDT seems to be a promising area of research in relation to anatomy instruction.

### Spiral curriculum, from simple to complex

Repetition with increasing detail as advocated by the students to enhance retention of knowledge appears to be in alignment with the characteristics of a *spiral curriculum*[38]: “A spiral curriculum is one in which there is an iterative revisiting of topics, subjects or themes throughout the course. A spiral curriculum is not simply the repetition of a topic taught. It requires also the deepening of it, with each successive encounter building on the previous one” [page 141]. Studies have confirmed that a spiral curriculum can be motivating because it activates and reinforces prior knowledge and stimulates a more advanced level of application and integration of knowledge and consequently increases expertise and feelings of competence [38,39]. In a spiral curriculum, knowledge is presented in a logical sequence from simple to complex. In the study of Smith & Mathias [40], students also reported that the amount of anatomy they needed to learn to be daunting. Controlled introduction of knowledge in the first loop of the spiral prevents students from being overwhelmed by details, one of the barriers to learning mentioned in the focus groups. Despite the attractiveness of the idea of a spiral curriculum, one may wonder how detailed anatomy instruction needs to be. Norman [41] has suggested that most medical specialists who rarely get to see inside the body may well get by with the simplified schematics in textbooks, or may even be advantaged by such an approach.

### Understanding, meaning and teaching in context

Our students were not the only ones to point to substantial differences between instruction in anatomy and in other biomedical sciences. In a study by Mattick & Knight [39], students suggested that anatomy was unique in that it represents a huge set of facts, codified in a specialised language and therefore demanded different learning strategies. We would suggest that because of the nature of anatomy, learning anatomy requires reliance on both surface and deep learning approaches. *Memorising* information is generally referred to as a *surface approach* to learning, in contrast to a *deep approach,* characterised by efforts to *understand* information by seeking a structure within the material and manipulating the information to make sense of it in relation to what is known of the subject matter [42]. Because of anatomy’s complex vocabulary, it has been hypothesised that a deep approach to anatomy learning needs to build on a preliminary stage of rote learning, which is difficult to distinguish from a surface approach [43,44]. The students in our study, however, perceived and described memorisation of details as an *endpoint* of learning rather than a stage in a process leading to understanding. Several other studies have reported similar findings [39,42,45]. It has even been reported that a surface approach to learning, finding anatomy learning daunting and not seeing the point of learning anatomy are associated [40]. One way to change students’ perceptions might be to adopt a strategy of assessment *for* learning, as opposed to assessment *of* learning, steering and fostering the learning of students and rewarding understanding and higher order cognitive skills [33,42].

Students appear to try and understand anatomy by giving meaning to what is learnt [22]. ‘To give meaning’ entails moving beyond labelling structures towards studying anatomy as an integrated whole. Contextualization of anatomy education, aimed at understanding relationships between anatomy and clinical manifestations (signs and symptoms) may make anatomy more meaningful. Furthermore it may increase their awareness of the relevance of and increase motivation to study anatomy. Considering that paper cases in tutorial groups were not perceived as effective in inducing students to study anatomy, we propose translating the PBL principle of contextual learning to anatomy education. A good example of such a strategy has been described by Scott [46], who created a case-based anatomy course for second-year medical students.

Students in this study specifically asked for help from teachers in achieving deeper learning, integration and application of knowledge. A similar view was expressed by Regan de Bere & Mattick [17]: “learning anatomy for clinical practice may well benefit from a subtle shift [from self-directed learning] to ‘directed self-learning’. [..] not suggesting […] that students should be spoon-fed, rather that self study should be guided by experts in the subjects of both anatomy and medicine” [page 582]. Such a shift would require teachers to carefully consider the content and delivery of laboratory sessions and lectures.

### Uncertainty

The results show that students feel very uncertain about their anatomical knowledge, especially at the start of clinical training. Their perceptions suggest that they are uncertain because they feel that their failure to master all the knowledge presented in the preclinical phase will get them into trouble during their clerkships: most importantly by losing face in front of physicians, fellow students and especially patients. Light [47] posited that uncertainty surrounding knowledge is one of five sources of uncertainty for medical students. Fox [48] described three types of uncertainty, including inability to judge whether problems encountered are due to lack of knowledge or other reasons. That our results also suggest that that senior students were able to handle uncertainty better than junior students could be a direct consequence of their clinical experience when their knowledge grows with subsequent training and they are increasingly exposed to, and learn, the main ways in which many physicians handle uncertainty [47,48]. These findings may also explain why recent graduates were more lenient than students in setting the required level of anatomical knowledge for beginning clerks [14].

Although it is one of the aims of PBL to ease the transition from preclinical to clinical learning, the results suggest that PBL was not successful in reducing students’ uncertainty and anxiety about their lack of basic science knowledge. It has been suggested that uncertainty can be mitigated by special training in which students are told that uncertainty is a normal part of medical training and practice and shown ways to deal with it (for example Kitto et al. [49]).

### Transfer of knowledge

Students indicated that it was difficult for them to apply the knowledge gained in the preclinical phase in clinical practice, for example in examining patients or interpreting a CT scan. Research has shown that anatomy and surgical teaching staff recognise, but have no formal educational strategy to overcome, this problem of transfer [50], that is, using a concept learnt in one context to solve a problem in a different context. It has been proposed that transfer should be the main focus of basic science education, although achieving retention is not unimportant as students need to remember information in order to be able to transfer it [51,52]. Strategies suggested by Norman [53] to enhance transfer, such as embedding a concept in a problem, encouraging active learning and mixed and distributed practice, would fit very well into a spiral PBL curriculum. A longitudinal experiment on the effectiveness of new methods for learning neuroanatomy described by Chariker et al. [54] is a good example of the type of further research that is needed to elucidate ways to attain (the combination of) learning, transfer and retention of anatomical knowledge.

## Conclusions

The results of this study appear to support the hypothesis that educational principles have a stronger impact on students’ perceived and actual anatomical knowledge than the educational approach underpinning a curriculum. Students’ perceptions indicated that a PBL approach in itself appears to be not enough to ensure adequate learning of anatomy. Change and innovation in teaching and assessment are required to improve anatomy education. A spiral curriculum, an increased focus on understanding of subject matter, contextualised instruction and strategies to enhance transfer all seem to hold promise for increasing students’ motivation, awareness of relevance, (retention of) knowledge and feelings of competence with regard to the learning of anatomy.

## Competing interests

The authors declare that they have no competing interests.

## Authors’ contributions

EB conceived and designed the study, participated in the data collection and analyses, discussed the results and drafted the original manuscript. AH and IV participated in data analyses, discussed the results and critically revised the original manuscript. AB discussed the results, and critically revised the original manuscript. AS made important contributions to the study design, participated in data collection, discussed the results and critically revised the original manuscript. CV made important contributions to the study design, discussed the results and critically revised the original manuscript. All authors read and approved the final manuscript.

## Authors’ information

EB (MSc) is a teacher at the Department of Anatomy of the Radboud University Medical Centre Nijmegen in Nijmegen, The Netherlands; and Ph.D. student at the Department of Educational Development and Research, Faculty of Health, Medicine and Life Sciences, Maastricht University, Maastricht, The Netherlands.

AB (PhD) is assistant professor at the Department of Educational Development and Research, Faculty of Health, Medicine and Life Sciences, Maastricht University, Maastricht, The Netherlands; and at the Institute of Psychology, Erasmus University Rotterdam, Rotterdam, The Netherlands.

AH (PhD) is a teacher at the Department of Anatomy/Embryology, Faculty of Health, Medicine and Life Sciences, Maastricht University, Maastricht, The Netherlands.

IV is a medical student at the Faculty of Health, Medicine and Life Sciences, Maastricht University, Maastricht, The Netherlands and research assistant for E.M. Bergman.

AS (MD, PhD) is a professor and Dean of Faculty of Health, Medicine and Life Sciences, Maastricht University, Maastricht, The Netherlands.

CV (PhD) is a professor and Chair of the Department of Educational Development and Research, Scientific Director of the School of Health Professions Education, Faculty of Health, Medicine and Life Sciences, Maastricht University, Maastricht, The Netherlands.

## Pre-publication history

The pre-publication history for this paper can be accessed here:

http://www.biomedcentral.com/1472-6920/13/152/prepub
